# Exploring the Challenges of Empathy in a Therapeutic Context: An Interpretative Phenomenological Analysis

**DOI:** 10.1177/10497323251331460

**Published:** 2025-04-28

**Authors:** Tracy Watson, Rebecca Waters, Lynelle Watts, David Hodgson

**Affiliations:** 1 School of Allied Health, 1649 Curtin University, Perth, WA, Australia

**Keywords:** empathy fatigue;, burnout;, affective empathy;, emotional distress;, compassion fatigue

## Abstract

Professionals working in therapeutic contexts are vulnerable to developing occupation-related stress conditions such as burnout, vicarious trauma, and compassion fatigue. In such contexts, empathy is accepted as a crucial component of practice. However, social neuroscience research suggests empathy may adversely impact workers’ well-being. While occupation-related problems like compassion fatigue have relevance, they do not fully explain the potential negative effects of empathy on worker well-being. Therefore, our study aimed to explore whether empathy posed a problem for those working in therapeutic contexts. Participants in this study were qualified professionals working in counselling roles under the Medicare Australia Better Access Initiative. Semi-structured interviews were conducted with fifteen participants, and data was analyzed using interpretative phenomenological analysis (IPA). This process led to the discovery of four superordinate themes: (1) Navigating Empathy: A Foundation of Practice and an Emotional Challenge; (2) The Interplay Between Therapists’ Lived Experience and Empathic Engagement; (3) Balancing the Demands of Empathy With Career Sustainability and Personal Well-being; and (4) When Values and Beliefs Diverge. Two subthemes are (1) Emotional and Physical Exhaustion and (2) Empathy—The Finite Resource. The findings indicate that while empathy is considered central to practice, various emotional, psychological, and occupational challenges are associated with its use.

## Introduction

Carl Rogers’s seminal work *Client-Centered Therapy* (Rogers, 1951) emphasized the pivotal role of empathy in fostering and maintaining effective therapeutic relationships. Rogers conceptualized empathy as a process in which the therapist immerses themselves in the client’s experiential world, describing this as “temporarily, living in his life, moving about in it delicately...communicating your sensing of his world as you look with fresh and unfrightened eyes at elements of which he is fearful” (Rogers, 1975, p. 4). Roger’s theoretical contributions have significantly influenced the disciplines of psychology, social work, and occupational therapy, establishing empathic engagement as a cornerstone of therapeutic practice (Watson et al., 2022). Although empathy is a contested concept, researchers generally agree that it involves some emotional connection with another’s experience (Watson et al., 2022). From a phenomenological perspective, Silani et al. (2013) suggest that empathy includes sharing emotions expressed by others and a cognitive process that distinguishes oneself from another. In social neuroscience, empathy has two distinct components: an affective state (experiencing others’ emotions) and a cognitive state (understanding others’ emotions) (Singer & Lamm, 2009). In this study, empathy is conceptualized as an affective experience, aligning with Svenaeus’s (2016) definition of it being “a perceptual-imaginative feeling towards and with the other person’s experiences. Empathy is made possible by affective bodily schemas, and it is enhanced by a personal concern for the target. Empathy is thus an emotional experience, but it nevertheless develops a cognitive understanding of the predicament of the other person” (p. 227).

Although empathy has gained widespread recognition for its potential to improve therapeutic outcomes (Cochran & Cochran, 2015), recent evidence suggests that empathy in therapeutic contexts may negatively impact workers’ well-being (Grynberg & Konrath, 2020; Klimecki & Singer, 2012; Leiberg et al., 2011). Specifically, functional magnetic resonance imaging (*f*MRI) research has demonstrated that affective empathy can activate the brain’s pain matrix, potentially leading to negative health outcomes (Decety & Lamm, 2006; Klimecki, 2015). For example, to investigate pain-related empathy, Singer et al. (2004) assessed *f*MRI data from couples while they were either experiencing direct physical pain (mild electric shock) or observing their partner receive the same painful stimuli. Their findings revealed empathy for pain-activated areas of the pain matrix, such as the anterior insula and anterior cingulated cortex, which are involved in processing the emotional components of pain. These findings are consistent with additional neuroimaging research demonstrating that brain regions involved in experiencing physical pain are similarly activated when observing or imagining another person in physical or emotional pain (Cheng et al., 2007; Lamm et al., 2011). This evidence highlights the presence of neural pathways in the brain that enable individuals to resonate with and understand others’ affective states, thereby fostering empathetic connections. Singer and Klimecki (2014) suggest that continual activation of empathy for pain responses may lead to empathic distress, defined as a “strong aversive and self-oriented response to the suffering of others, accompanied by the desire to withdraw from a situation in order to protect oneself from excessive negative feelings” (p. R875). Klimecki and Singer (2012) contend that individuals in helping professions are especially vulnerable to empathic distress due to their repeated exposure to the suffering of others. Over time, this can result in a diminished capacity for empathetic engagement and increased negative emotional states. To more precisely capture the nature of emotional exhaustion and burnout experienced by helping professionals, Klimecki and Singer (2012) introduced the term empathic distress fatigue, which they argue more accurately reflects this phenomenon.

Stebnicki (2015) coined the term empathy fatigue, highlighting its conceptual distinction from related phenomena such as compassion fatigue and burnout. Stebnicki (2015) states that empathy fatigue is the exhaustion of empathic resources from prolonged exposure to clients’ emotional pain and suffering. Unlike burnout and compassion fatigue, which encompass broader occupational or caregiving stressors, empathy fatigue is specifically associated with the emotional demands of sustained empathic engagement (Stebnicki, 2007). Stebnicki (2015) further observes that professionals who employ empathic approaches, particularly those in the early stages of their careers, are vulnerable to this phenomenon. Symptoms of empathy fatigue are similar to those of depression and anxiety, with contributing risk factors including poor support networks and maladaptive coping styles (Stebnicki, 2007). These risks underscore the need to investigate empathy fatigue further to protect helping professionals’ well-being.

The helping profession literature recognizes that workers are at risk of developing occupation-related stress conditions such as burnout (Schaufeli et al., 2009), vicarious trauma (McCann & Pearlman, 1990), and compassion fatigue (Figley, 2002). Although these concepts are often used interchangeably, they have distinct differences. Burnout was popularized by Freudenberger as a clinical condition in the mid-1970s, emphasizing its prevalence across various professions, particularly high-stress work environments that often lack effective management strategies (Schaufeli et al., 2009). Burnout impacts individuals’ personal and professional well-being, encompassing three key dimensions: emotional and physical exhaustion, depersonalization (detachment from others), and a reduced sense of perceived competence in one’s professional role (Brotheridge & Grandey, 2002). In the context of counselling, Yang and Hayes (2020) identify three categories of therapist-specific factors that predict burnout: (1) the work environment, workload, and availability of support, (2) the therapist’s mental health history, confidence in their professional abilities, and personality traits such as emotional intelligence, and (3) the nature of the client’s presenting issues, as well as their level of commitment to the therapeutic process. Prolonged stress linked to burnout has been shown to affect areas such as attention and executive function, underscoring this condition’s broader psychological and neurological impact (Arnsten & Shanafelt, 2021). Stress triggers the release of hormones such as cortisol and adrenaline, enabling the body to cope with short-term difficulties. Prolonged activation of this response may adversely affect the brain and other bodily organs (Schauer & Elbert, 2010). For example, Arnsten and Shanafelt (2021) found that unmanaged stress impairs the prefrontal cortex (PFC) functioning, a region responsible for the top-down regulation of thoughts, actions, and emotions. In high-stress environments, such as healthcare settings, chronic stress can lead to structural damage and loss of connections in the PFC (Arnsten & Shanafelt, 2021). Schaufeli et al. (2009) suggest this loss undermines individuals’ ability to effectively emotionally self-regulate, potentially explaining the link between burnout and avoidant behaviors, such as withdrawing from challenging situations or responsibilities as a maladaptive coping mechanism.

McCann and Pearlman (1990) introduced the concept of vicarious trauma, asserting that the experiences of professionals working with trauma survivors lead to changes in the therapist’s fundamental beliefs about themselves and others caused by their exposure to clients’ traumatic experiences (McCann & Pearlman, 1990). Building on this foundation, Figley (2002) introduced compassion fatigue within the helping profession literature, asserting that “the very act of being compassionate and empathic extracts a cost under most circumstances. In our effort to view the world from the perspective of the suffering, we suffer” (p. 1434). Figley (2002) has identified empathy as a foundational element underlying compassion fatigue, highlighting its centrality to this phenomenon. Recently, compassion fatigue has faced scrutiny within fields such as nursing (Sinclair et al., 2017) and medicine (Fernando & Consedine, 2014), where it has been criticized as a poorly defined construct. For instance, Ledoux (2015) contends that compassion fatigue “rests on a most fragile foundation” (p. 2016), while Sinclair et al. (2017) assert that the concept “has largely been unchallenged and remains poorly understood” (p. 20). Advances in neuroscience have further complicated the understanding of compassion fatigue. Studies using *f*MRI have revealed distinct neural pathways for compassion and empathy (Singer & Lamm, 2009). Affective empathy activates the pain matrix, whereas compassion, defined as having concern for another’s suffering coupled with a desire to alleviate it, is associated with activating dopaminergic and oxytocin pathways and is linked to affiliation and reward (Goetz & Simon-Thomas, 2017). These findings suggest that compassion may promote emotional well-being, whereas empathic distress, rather than compassion, is potentially the primary source of challenges faced by helping professionals (Klimecki & Singer, 2012).

Burnout, vicarious trauma, and compassion fatigue have received significant attention within the helping profession literature, highlighting the vulnerability of practitioners to occupation-related stress conditions. Bride (2007) highlights that professionals supporting traumatized individuals are particularly susceptible to these stress-related challenges due to the profound emotional and psychological demands inherent in their work. In Bride’s (2007) study, 70% of social workers reported experiencing at least one symptom of secondary traumatic stress, 55% met the criteria for at least one symptom of post-traumatic stress disorder (PTSD), and 15% met the criteria for a PTSD diagnosis. Similarly, Figley (2002) cites research conducted in Australia, where 54.8% of surveyed mental health professionals reported distress, and 35.1% described themselves as “extremely emotionally drained” (p. 1435). Another study by Craig and Sprang (2010) found that 5% of clinical social workers and psychologists scored as being at high risk of compassion fatigue or burnout. Historical perspectives on the emotional costs of helping work offer additional insights. Rogers appeared to recognize that fatigue syndromes can affect counsellors, emphasizing their need to restore their minds, bodies, and spirits following prolonged psychotherapy sessions (Kirschenbaum, 2007; Rogers, 1980). More recently, authors such as Zeman and Harvison (2017) highlight the urgent need for “educators, students, and practitioners to invest in strategies to combat burnout” (p. 1). Similarly, Lloyd et al. (2004) suggest “that the focus of future research should be on identifying characteristics of mental health work that contribute to emotional exhaustion” (p. 752), while Fahy (2007) suggests that “the unspoken question is why workers leave the field and part of the answer may lie with the stress and strain of empathic work” (p. 199).

Beyond these stress-related conditions, emotional labor offers further insight into the challenges faced by helping professionals. Hochschild (1983) defines emotional labor as regulating one’s emotions to meet workplace expectations. This process often requires surface acting (modifying and controlling emotional expressions) or deep acting (controlling feelings to match organizational expectations) (Brotheridge & Grandey, 2002). For therapists and other helping professionals, this may involve maintaining empathy while managing emotional responses during challenging client interactions. Prolonged emotional labor, particularly in the absence of adequate support or coping strategies, has been associated with emotional exhaustion, a central component of burnout (Brotheridge & Grandey, 2002).

Despite the significant attention given to burnout, compassion fatigue, and vicarious trauma, further research is warranted to help deepen our understanding of the potential negative effects of empathic practice in therapeutic contexts. Exploring the complex interplay between emotional engagement and occupational well-being will provide professionals with better insights and strategies to manage empathic distress, burnout, and emotional fatigue.

## The Present Study

This study forms part of a broader PhD research project investigating how professionals in therapeutic contexts used and described empathy and whether they experienced adverse effects related to its application. While existing literature highlights that empathic engagement can lead to emotional exhaustion, referred to as empathy fatigue, much of this research is grounded in social neuroscience rather than first-person accounts. To address this gap, the present study used a qualitative design to explore helping professionals’ experience of empathy in therapeutic settings.

## Methodology

Interpretative phenomenological analysis (IPA) was chosen for this study as IPA focuses on the detailed examination of a phenomenon and lived experiences (Smith et al., 2022). In this study, we focused on the concept and experience of empathy within a therapeutic context. The phenomenological approach allowed for collecting and analyzing rich and detailed information, unrestricted by predefined categories such as those found in survey studies. Further, IPA emphasizes the use of the hermeneutic circle, enabling an examination of how the phenomenon emerged while supporting various ways of thinking about the data. This process aided an understanding of what was unique for each participant, the convergence and divergence between the sample, and the relationships with pre-existing knowledge. Homogeneity of the sample is important in IPA because studies are framed around understanding a phenomenon within a specific context (Smith et al., 2022). This study focused on helping professionals in counselling roles where empathy is an accepted practice component, ensuring the participants had a direct experience of the phenomenon.

### Sampling Method

Participants worked in private counselling practice, delivering Focused Psychological Strategies under the Medicare Australia Better Access Initiative, thus ensuring homogeneity. Medicare Australia is a publicly funded health insurance program that aims to provide all Australians equitable access to health services. The Better Access Initiative was introduced in 2006 to enhance mental health treatment within the community (Australian Government Department of Health and Aged Care, n.d.). Under this scheme, eligible professions are social work, occupational therapy, and psychology. Social workers must be registered as mental health social workers with the Australian Association of Social Workers (AASW). Occupational therapists must hold membership with OT Australia and possess a minimum of 2 years of supervised mental health practice. Psychologists must be registered with their respective State or Territory Psychologists Registration Board (Australian Psychological Society, 2019).

### Recruitment

Participants were recruited through professional networks such as the Australian Association of Social Workers, Occupational Therapy Australia, and the Psychologists Registration Board. A request was made to distribute information about this study to their members, and interested individuals contacted the lead researcher (TW) to confirm eligibility. A snowballing technique was also employed.

### Data Collection

Eligible participants attended one semi-structured interview with TW, during which they were asked open-ended questions. COVID-19 restrictions meant that the interviews were conducted via Microsoft Teams, being approximately 45–60 minutes in length. The interviews were voice-recorded and later transcribed by TW. Each participant was sent a copy of their respective transcript for review, allowing them to contribute additional information if necessary. No changes were made. Four interview questions were included in the larger study: (1) Can you tell me about any personal experiences that influenced your decision to work as a SW/OT/psych? (2) When thinking about the concept of empathy as it relates to your counselling role, how would you describe it? (3) In what ways do you use empathy in your practice? (4) Have you experienced any challenges using empathy in your practice? This paper focused on analyzing and presenting results from question 4. We determined that this data was most significant for dissemination in a published article. The findings from the other three questions were thoroughly documented and elaborated upon in separate chapters of the corresponding PhD thesis.

### Ethics

This research project is a partial fulfilment of the requirements for a larger PhD study that received ethics approval from the Curtin University Human Research Ethics Office and was conducted following the National Statement on Ethical Conduct in Human Research (National Health and Medical Research Council, the Australian Research Council, and the Australian Vice-Chancellors’ Committee, 2007). Participation was voluntary, and consent was obtained in writing. No ethical issues arose during this study. All data was de-identified to protect the confidentiality of the participants, and each participant was assigned a pseudonym.

### Trustworthiness

Smith’s (2011) criterion for a “good” study guided this research project’s trustworthiness. Following Smith’s (2011) instructions, findings are presented so that the researcher’s (TW) interpretations are easily identifiable and supported by verbatim transcript excerpts. Rodham et al. (2015) explain that IPA “researchers involved in the analysis should listen to the audio recordings; failure to do so increases the potential for researchers to superimpose their own presuppositions or interpretative bias onto the data” (p. 59). As such, TW listened to the recordings throughout the analysis to address any potential bias and, thus, risk to the trustworthiness. Furthermore, TW kept reflective notes detailing thoughts and feelings about the research process, which were discussed and addressed with the other authors.

### Data Analysis

1. *Reading and re-reading*

TW conducted the interview analysis following the approach outlined by Smith et al. (2022). The phase involved active and immersive engagement with the data. This foundational step provided the groundwork for the subsequent stages of analysis.

2. *Exploratory noting*

This second step examines how participants talk, understand, and think about the interview questions (Smith et al., 2022). The interviews were re-listened, and the transcripts were reviewed multiple times. During this process, observations, reflections, and initial thoughts were documented. Particular attention was paid to the participant’s sense-making, including their use of language such as metaphors, distinctive phrases, and intonation.

3. *Constructing experiential statements*

In the third step, the analytical focus shifts away from the transcript (Smith et al., 2022). Adopting a reductionist approach, this process involved transforming the notes into experiential statements. These statements captured the participant’s experiential claims and understandings while integrating the researcher’s interpretations.

4. *Connecting experiential statements*

This process entails identifying patterns within the experiential material (Smith et al., 2022). The primary objective was to construct a coherent framework that captured the essence of the participant’s experiences.

5. *Personal experiential themes*

In this stage, the connected, experiential statements are transformed into “provisional” personal experiential themes (PETs) (Smith et al., 2022). This process informed a higher level of abstraction, resulting in concise interpretive summaries for each interview. To ensure a strong evidentiary base, relevant quotes from the transcript were incorporated under each PET, reflecting the participant’s words and the researcher’s interpretations.

6. *Cross-case analysis—Group experiential themes*

After repeating steps 1 through 5 for each transcript, this step involved identifying patterns of similarity and difference across the PETs, producing a set of group experiential themes (GETs) (Smith et al., 2022). These GETs highlighted shared and unique experiences rather than just a group norm. A cross-case analysis developed four GETs and two subthemes. Each theme is supported by succinct summaries and participant statements that address the interview question: *Have you experienced any challenges using empathy in your practice?* To improve the readability of the participant’s statements, irrelevant or repeated words were removed. Ellipses (…) indicate omitted or inconsequential text and merged sentences, while square brackets ([]) denote words added by the researcher, TW, for context or clarity that were not part of the participant’s statements.

## Results

Fifteen participants were interviewed for this study, three male and 12 female. Four of the participants were psychologists, four occupational therapists and seven social workers. The average age of the participants is approximately 51.5 years, and the average years of experience in their respective professions are approximately 13 years for psychologists, 25.2 years for occupational therapists, and 25.2 years for social workers. The average time working in a counselling role for the participants is approximately 10.5 years.

### Theme 1: Navigating Empathy: A Foundation of Practice and an Emotional Challenge

This overarching theme underscores the participant’s nuanced understanding and lived experiences of empathy in therapeutic practice. Their reflections illuminate its centrality to their practice, the emotional challenges its use entails, and the importance of reflexivity in managing these challenges. Rebecca’s narrative, reflecting the experiences of the other participants, positions empathy as the cornerstone of practice: “It’s the make or break to be able to really be in there with them.” Similarly, Casey conveys its foundational role in forming the therapeutic relationship*:* “Empathy is the critical component … and so, to me, it is the primary tool of developing a therapeutic alliance.” Other participants like Kate viewed empathy as a foundational element underpinning other therapeutic techniques: “It’s something that sort of underpins the other work that you do.” Tanya expands on this perspective, perceiving it as the foundation upon which counselling strategies are applied: “If you don’t have that empathetic connection … then it makes the application of all that other stuff harder, or pointless.” Despite its foundational importance, participants also recognized empathy as being dual-edged. Rebecca’s metaphor captured this duality, illustrating both its capacity to foster deep connection and its potential to become an emotional burden: “… I think it can be a two-edged sword … it’s like an emotional weight that can gradually weigh you down over time. It’s almost like an emotional bombardment when you empathize.” Further, the participant’s accounts highlight the profound emotional labor inherent in empathic engagement and their efforts to manage this. For instance, Tanya’s use of “healthy dissociation” reflects her effort to balance empathic engagement and emotional self-regulation. She describes moments of zoning out during sessions as a coping mechanism for overwhelming content, while her resolve to “come back and reconnect” underscores her dedication to being present:

Lots of times, I have caught myself in terms of that problem of empathy; there are lots of times where I just zone out in session with a client, which I would probably call healthy dissociation because I’m so overwhelmed by all the content that’s coming up and like, Oh, my goodness, I’ve just not been listening, let me come back and reconnect.

Jake offers a candid acknowledgment of empathy’s toll on his well-being: “Well, it does. I know it does, and I know that it has … If you are constantly in and out of that process, then it’s got to take its toll.” His term “empathetic overload” further conveys the emotional strain that empathic engagement can impose, adding, “Empathy can actually be quite toxic.” Similarly, Rose reflects on her emotional vulnerability in response to distressing situations: “Quite often, I will fill up; I can feel my eyes filling up. It’s like I’m not sure what to do here.” To manage these moments, she shared her strategy of temporarily leaving the room to process her emotions privately: “I’ve had to go to the kitchen or go into the toilets and cry to let it out because it’s impacted me emotionally. If I can’t hold it back in the room.” These accounts collectively demonstrate that while empathy facilitates connection and understanding, it also has the potential to become a source of emotional strain. The participant’s narratives further reveal a delicate balancing act, requiring reflectivity and awareness to sustain their therapeutic work.

This interplay is further illuminated in participants’ accounts of burnout, which they described as a consequence of sustained empathic engagement. Rebecca succinctly encapsulates this relationship: “Empathy can lead to burnout.” Similarly, Paul explains, “There’s some level of burnout going on … and I find that quite shocking.” His mention of the term “empathic distress” also highlights the emotional toll of engaging with clients’ suffering and the importance of emotional regulation to prevent burnout: “… Empathic distress can lead to burnout if we’re not kind of regulating and … in a space that is actually assisting … I think that will lead to burnout.” John’s narrative reveals his emotional commitment: “After working with brain-injured clients and really investing … you are part of the family and embedded in the family … so empathy is really an emotional investment,” and the cumulative impact of this on well-being. His resigned response, “Did you experience burnout?” [Interviewer], “Yep, not coping with this anymore” [John], reflects the toll of prolonged emotional engagement. Kate offers a different perspective by emphasizing the protective role of client diversity in mitigating burnout. She explains that engaging solely in grief or trauma work would likely exceed her emotional limits: “Grief-related things … would lead to a bit of burnout for me … just doing 100-percent trauma work. I think that would be maybe a bit too much.” Her emphasis on variety in presenting issues suggests that diversity reduces emotional strain and prevents exhaustion. Kate connects burnout to both physical and mental health, illustrating its broader impact: “That’s where you can become either physically unwell or just mentally exhausted; that’s where it impacts empathy.” Tanya provides a nuanced perspective, framing empathy as “… that felt sense that you get when you’re with a person.” While stressing the importance of this connection, she also acknowledges the risks: “… We also need to be kind of taking care of ourselves because … we can kind of get distressed or burnout … have all that fatigue.” Here, Tanya’s narrative vividly captures the intensity of her empathic engagement and the abrupt, impactful nature of burnout: “I think it’s what causes … burnout; it’s your foot on the accelerator, and then all of a sudden, you hit a brick wall.” The metaphor of the “accelerator” powerfully conveys the visceral, embodied experience of burnout. To manage this, Tanya stresses the importance of finding a balanced approach: “… Learning a middle path, which means being aware of both polarities … if there is an extreme polarity for empathy, we call it sort of pathological altruism.” Her reference to “pathological altruism” exposes the potential harm of excessive empathy and underscores the necessity of intentional emotional regulation to sustain well-being. Paul’s reflections similarly highlight the risks of unchecked emotional engagement. He admits moments where his emotional responses disrupt the therapeutic process: “I’ve hijacked the experience, and it’s like I know what you’re saying … instead of offering that safety, I’m kind of up-regulating in the same way as the client is.” His recognition that “we’re both a little bit off the rails … the chance to co-regulate, it’s kind of gone” demonstrates the relational nature of empathy and the need for reflexivity in practice.

#### Subtheme 1: Emotional and Physical Exhaustion

This subtheme builds on participants’ reflections, exploring the cumulative demands of empathic engagement and its toll on their well-being. Their accounts reveal the profound emotional and physical exhaustion, often leaving them feeling drained and depleted.

Dianne describes the energy-intensive nature of empathy, noting how it “Requires, or takes or drains” her energy. She reflects on the struggle to manage this emotional toll, likening it to watching a “dark, heavy” movie that leaves her feeling exhausted:

… It can be really tiring and really exhausting. You can feel like you’ve been through; it’s just like you turn on a movie, and it’s particularly dark, heavy, and at the end, you feel a bit, oh, God, I’m done … sometimes it is really wearing, and tiring.

Kim’s narrative highlights the progression of empathic fatigue, distinguishing it from physical tiredness. She articulates the emotional effort involved: “… Holding space, the work of being able to bear with another someone’s suffering, it’s tiring work,” describing this as unique to empathic labor: “… Empathy is exhausting; I feel absolutely exhausted … quite different from a hard day’s work in the garden.” Furthermore, she explicitly highlights that even in the absence of vicarious trauma, her work is draining: “I don’t feel like I’m being vicariously traumatized … but actually, at the end of a really big day full of people’s trauma, there is a level of fatigue.”

Casey’s account illustrates how emotional exhaustion can affect therapeutic composure. She candidly recounts moments when fatigue affected her professional responses, underscoring the importance of recognizing and addressing early signs of emotional depletion:

When I’m really tired, when I’m emotionally exhausted, if I am then speaking to a long-term client who is struggling to deal with something that we’ve been working on for a long period, then I just want to go … do what I tell you to do …, occasionally that comes out my mouth, but it was a bad thing because it was to me not acting therapeutically, it was me losing it. And I’m careful to feel the signs of that happening.

These narratives provide insight into the significant toll of sustained empathy, emphasizing this exhaustion’s unique and cumulative nature. Their experiences further highlight the necessity for strategies to mitigate fatigue, enabling practitioners to maintain their capacity for effective, compassionate care. Their narratives further revealed that empathy use has limitations.

#### Subtheme 2: Empathy—The Finite Resource

The subtheme examines the participant’s characterization of empathy as a finite resource, further underscoring the emotional toll of sustained empathic engagement and the strategies used to manage its depletion. Casey likened the experience of emotional fatigue to “when you get to the bottom of your tank,” metaphorically illustrating how prolonged empathy can hinder professional effectiveness. She noted that the emergence of “a little tiny hint of resentment” serves as an early warning of exhaustion. For Casey, the finite nature of empathy underscores the importance of actively monitoring emotional and cognitive reserves: “Your emotional energy gets depleted. You need emotional energy to empathize; it’s limited; it’s not a limitless tank.” Kate echoed this sentiment, emphasizing the need for strategies to replenish her emotional reserves. She described nurturing empathy through boundary-setting and transitional strategies that create separation between work and personal life:

For myself, anyway, it does deplete, and I need to nurture it, really, to keep it going … I’m fairly good at separating work from home. I have a bit of a drive home, and I’m able to listen to a podcast and sort of separate that.

Kate’s proactive approach reflects her commitment to maintaining balance to sustain her empathic capacity in the long term. In contrast, Jade offered a different perspective, describing empathy as an “infinite kind of source.” While this suggests that empathy is intrinsic and ever-present, she acknowledged variability in her ability to access it, linking this to self-care practices: “I don’t think that I really run out of it; it’s kind of always there … My capacity to access it might vary with how well I’m looking after myself.” Jade’s perspective adds complexity to the theme by suggesting that while empathy itself may not be exhaustible, its accessibility is contingent on individual well-being.

Overall, these themes illustrate the centrality of empathy in therapeutic practice, highlighting its dual-edged nature as both a cornerstone of therapeutic connection and a source of emotional strain. The participants’ insights emphasize the importance of balance and strategies to manage empathic engagement, ensuring effective practice and personal well-being.

### Theme 2: The Interplay Between Therapists’ Lived Experience With Empathic Engagement

This theme explores how participants’ personal histories, while enhancing their empathic abilities, also present emotional challenges. Several participants shared how their own difficult experiences could evoke strong feelings during therapeutic interactions. Jake reflected on the influence of his childhood struggles, saying, “My own trials and the school of hard knocks … taught me a lot about helping those going through similar kinds of issues.” Megan offered a more explicit connection between her personal experiences and her therapeutic work: “I felt like I could really relate to people suffering from depression and anxiety … for me that the lived experience … you show people that you can relate through your own experience.” Amy also identified how her past enhanced her understanding of clients: “I’ve had lots more experiences, I understand my clients, … So, I guess, yes, my own lived experience.” However, participants also noted that their personal histories could elicit heightened emotional responses in certain situations. For example, Megan described feeling a stronger emotional resonance when clients’ struggles mirrored her own: “I probably feel the emotion more when they’re going through something similar, and that’s just the reality.” Similarly, Amy recounted instances when parallels between her past and her clients’ situations triggered an emotional response:

There are times when I will probably have that emotive response, which would be more about my emotive response. For example, I had some challenging years with my daughter when she was a teenager … and I had some young people sort of similar age to her come in and talk about their relationship with their mother … that’s where it’s triggering in me.

Amy acknowledged feeling anxious during these moments, reflecting on the emotional labor required to manage such challenges: “I think anytime, where I noticed that I might feel a little anxious … that it’s certainly more triggering my own experience in me.” Her statement: “There is that experience in the room,” further illustrates an awareness of how her past shapes her therapeutic interactions. John’s reflections further highlight the influence of personal history on empathy. As a father, he described heightened emotional engagement when working with parents facing similar challenges: “That affect with parents has been a lot more acute … like if they’re sick or had to go to hospital, it’s almost like a triggering of your own emotions, I do know what you’re feeling.” John’s use of the word “acute” emphasizes the intensity of his responses, while his description of being triggered reflects the relational nature of empathy. His personal experiences enhance his ability to connect with clients and foster understanding, but they also evoke strong emotional reactions.

Overall, this theme reveals the complexity of empathic engagement. While the participants’ personal histories deepen their capacity for connection and understanding, they also evoke emotional challenges, particularly when their past closely aligns with their clients’ struggles.

### Theme 3: Balancing the Demands of Empathy With Career Sustainability and Personal Well-Being

This theme explores participants’ reflections on managing the tension between career sustainability and personal well-being. Many described considering significant lifestyle changes, such as reducing work hours or changing careers, to cope with the emotional strain of empathic engagement.

Jake’s comments highlight the importance of self-care in maintaining professional sustainability, noting the consequences of neglecting it: “It’s got to deplete something if you don’t acknowledge and take care of yourself.” He shared how moments of depletion led him to reassess his career choices: “Do something different, work for Uber, or something like that. You’ve just got to know this is not working for me, and I’m not looking after myself, I’m becoming depleted.” Jake’s contemplation of alternate work reflects a broader effort to safeguard his well-being by re-evaluating his work environment and boundaries. Dianne echoed similar concerns, admitting that emotional overload sometimes diminished her ability to empathize: “… Sometimes I do find it difficult to empathize, but I think that is normally around times when I have had too much, and I’m thinking, what else can I do with my career?”. Her struggle highlights the profound impact of empathic fatigue on her professional identity and long-term aspirations.

Other participants shared similar turning points. John, for instance, described reaching a breaking point in his career due to emotional exhaustion: “I just kind of went, yeah, that’s me done … yep, not coping with this anymore.” He ultimately chose a less emotionally demanding job: “I ended up mowing lawns for a year.” This decision underscores the importance of stepping back from emotionally taxing roles to protect one’s mental health. Similarly, Paul recognized the cumulative strain of empathic engagement, stating, “This is not the work that I want to do.” Paul’s reflection marks a gradual yet significant process of re-evaluating his career to prioritize his well-being: “I can see myself becoming quite unwell if nothing changes.” Megan’s narrative further illustrates the challenges of maintaining emotional balance in demanding roles. She candidly described her struggle with burnout: “I feel tired … I feel burnt out … I’m not sure how much longer I can do this work; I’ll be really honest with you.” Her acknowledgment of the strain this work places on her well-being reflects the internal conflict of balancing professional commitment with self-care. As such, she reconsidered changing her profession in light of such experiences. For Kim, the emotional demands of her work extended to her personal life, affecting her relationship with her partner: “It was often hard for me to really attend to my partner’s needs for attention.” Consequently, Kim decided to work part-time: “I only work part-time now.” This adaptive response illustrates her proactive approach to managing her emotional resources while maintaining a professional balance. Rebecca described how the emotional intensity of empathy could lead to protective detachment: “Defence systems start to come up, trying to protect yourself, and when you become aware of that, and that’s kind of when it does affect your empathy.” While this detachment served as a temporary coping mechanism, Rebecca acknowledged its potential to hinder her empathic engagement. Her reflection: “Having that insight to go, whoa, why do I do this?”, captures a moment of questioning her professional path.

These narratives collectively highlight the tension between participants’ capacity for empathy and the emotional costs of their work. While their reflections underscore the challenges of empathic engagement, they also reveal the strategies participants employ to protect their well-being and sustain their professional identities.

### Theme 4: When Values and Beliefs Diverge

This theme explores the boundaries of empathy, particularly when participants encountered clients whose beliefs or behaviors contrasted with their own. Megan’s account illustrates this conflict when clients’ values diverge from hers. She reflected on finding it “very challenging” to maintain empathy while struggling to connect emotionally with a client engaged in conspiracy theories. This underscores how ideological divergence can create barriers to emotional connection. Megan’s reflections further highlight her self-awareness and professional dedication, as she consciously maintained compassion and therapeutic rapport despite her internal struggles:

… It’s normally when a client’s values or beliefs are completely at odds with my own. So, for example, I’ve got one client who’s been engaged in a conspiracy theory, and I’ve found it very challenging … when I reflect on that, I have been able to demonstrate compassion and therapeutic rapport with her, but internally, I’ve really struggled to generate a feeling.

Similarly, John described the limits of empathy when working with clients whose actions evoke personal discomfort, such as those involved in child exploitation. While he views empathy as “almost unconditional,” he acknowledged a shift from affective to cognitive empathy in such situations, using professional skills to maintain engagement while maintaining his emotional distance:

Empathy almost feels unconditional, up until a point, but with clients that have been into child exploitation material and stuff like that. I think maybe that’s when it goes from an affective to a cognitive state ... I’m trying to remain empathetic to how the person got here … that’s when the cognitive skills step in or take over; you can still go through the skills but appear to be empathetic when, in fact, you’re actually just being professional and using the skills as opposed to a genuine kind of emotional connection with the person.

In contrast to Megan and John, Tanya and Leah opted to disengage from client groups they found particularly challenging. Reflecting Tanya’s ethical commitment to professional integrity and self-preservation, she explained her decision as a way to avoid offering “a fake kind of empathy,” which she believed could undermine the therapeutic process:

I have recognized in my own practice that there are some client groups that I just really struggle to build empathy with, and I will kind of, I’ve chosen to not work with those groups at the moment … it’s a fake kind of empathy, so, I guess, you know, on a meta-level, that is being empathetic towards those clients by not subjecting them to working with me.

Leah shared a similar experience, reflecting on her discomfort with a specific client and her decision to limit engagement. Her decision, driven by her inability to empathize effectively, reflects an ethical stance to protect both her and the client:

I just felt really uncomfortable around him … so, it was something I found really challenging because of what I knew about him … I didn’t follow up with him as vigorously as I might have someone else because I didn’t want him to say he wanted to come back to me ... I didn’t want to support him. That is the bottom line.

Overall, this theme illuminates how personal values and beliefs can create boundaries to empathic engagement. Participants’ reflections reveal a dynamic process of navigating professional obligations and personal boundaries. While some adapted by shifting their approach to empathy, moving from affective to cognitive, others made ethical decisions to disengage, highlighting empathy’s multifaceted and context-dependent nature in practice.

## Discussion

The primary aim of this study was to explore the challenges experienced by professionals working in therapeutic contexts when using empathy in their practice. While empathy was considered an important component of therapeutic practice, the findings reveal that participants faced various emotional, psychological, and occupational challenges with its use. Notably, while they did not explicitly mention experiencing compassion fatigue or vicarious trauma, they identified burnout as a significant issue associated with empathy use. Although Freudenberger found no direct link between empathy and burnout, he considered burnout a serious occupational hazard, particularly in helping professions (Schaufeli et al., 2009). Previous research on the relationship between empathy and burnout has yielded mixed results. Some studies suggest that vicariously experiencing others’ emotions may increase the risk of burnout, while others propose that empathy can be a protective factor against it (Altmann & Roth, 2021; Stosic et al., 2022). Interestingly, Palumbo et al. (2024) found that sustained empathic engagement often served as a risk factor, contributing to exhaustion, diminished well-being, and burnout, even though clinicians’ use of self-empathy had a protective effect.

In the present study, participants reported that unregulated emotional engagement through empathy could adversely impact their well-being, ultimately impeding their ability to support clients effectively. This finding is consistent with previous research, such as Gleichgerrcht and Decety (2013), who found that physicians struggling to regulate their negative affective arousal were more susceptible to emotional exhaustion, detachment, and a diminished sense of accomplishment. In line with this, the participants further highlighted the importance of managing emotional responses to prevent emotional distress and burnout. These responses were often heightened when clients shared similar life experiences with the participants.

The benefits of using lived experiences to connect with and understand others in therapeutic settings are well documented (Cochran & Cochran, 2015). In this study, participants noted that drawing on their lived experiences enhanced their ability to empathize with clients’ situations. However, they also recognized that such experiences could elicit strong emotional responses. Several emphasized the importance of monitoring their emotions during therapy sessions to avoid distress, particularly when working with clients who shared similar or traumatic experiences. Exposure to certain client presentations, such as grief and trauma, was especially challenging for some. This result aligns with prior research, which has found that close emotional engagement can lead to personal distress, particularly when grounded in shared life experiences (Grynberg & Konrath, 2020). While lived experiences can be valuable in therapeutic contexts, this study highlights potential challenges. Specifically, the findings confirm that emotional overlap, particularly when empathizing with others’ negative experiences, may contribute to personal distress, a phenomenon linked to the affective components of empathy (Davis, 1980). Affective empathy activates distinct neural networks like the amygdala (Grynberg & Konrath, 2020). Amygdala activation triggers the release of stress-related hormones, including cortisol and adrenaline. Prolonged or repeated activation of these systems is associated with chronic stress and can lead to exhaustion. This occurs through the physiological arousal or fight–flight response it induces, which elevates heart rate, blood pressure, respiration, and muscle tension (Shanafelt, 2021). While such responses may assist individuals in coping with immediate emotional challenges, sustained activation can deplete energy reserves and adversely affect health and well-being over time (Schauer & Elbert, 2010). For example, sustained or intense stress and negative affect can significantly increase the risk of developing mental health disorders, including depression and anxiety (Kring, 2008). These insights underscore the need for strategies to mitigate the risks associated with emotional distress and depletion in therapeutic practice.

The metaphor of burnout, likened to the extinguishment of a fire, illustrates the depletion of emotional energy, highlighting the need for consistent replenishment to sustain functionality (Craig & Sprang, 2010). This framing positions burnout as a late-stage phenomenon, emphasizing the importance of proactive prevention and mitigation strategies. Similarly, participants in this study described empathy as a finite resource, vulnerable to exhaustion through prolonged emotional engagement. One participant encapsulated this through the vivid metaphor of reaching the bottom of their empathy tank, illustrating the tangible impact. Participants stressed the importance of managing the energy demands of empathic engagement to prevent physical and emotional exhaustion. This relationship between empathy and exhaustion aligns with Stebnicki’s (2015) concept of empathy fatigue, which describes the depletion of empathic resources caused by sustained exposure to others’ emotional pain. Furthermore, the findings echo Figley’s (2002) framework of compassion fatigue, which identifies the cumulative toll of ongoing compassionate and empathic engagement, often manifesting as stress, anxiety, and emotional exhaustion. The present study also identified strategies employed by the participants, such as emotional regulation, detachment, and self-care, to mitigate the risks associated with sustained empathic engagement. However, their limited efficacy highlights the critical need for systemic and organizational interventions to provide more robust support. For example, one participant described moments of losing the ability to empathize, which they believed diminished their effectiveness as a therapist and even led them to consider a career change. Others also expressed similar concerns about the long-term sustainability of their roles, prompting lifestyle adjustments such as taking time off or transitioning to part-time work. In contrast, one participant offered an alternative perspective. While framing empathy as an inexhaustible resource, it is noted that its accessibility fluctuated based on self-care practices. This perspective is corroborated by the ideas of Figley (2002), who suggests that while empathy may be enduring, the capacity to express it relies heavily on maintaining physical and emotional well-being.

The participants also described instances where empathy was constrained by their ideological or moral conflicts, revealing the boundaries of empathic engagement. While empathy fosters emotional connection, its moral role is debated across disciplines (Prinz, 2011). Research indicates that empathic understanding diminishes when individuals perceive others as considerably different (Echols & Correll, 2011; Fowler et al., 2021). In this study, participants responded to such conflict in different ways. Some chose to stop supporting clients, suggesting that the conflict was significant enough that they recognized it hindered their ability to provide effective care. Others continued but shifted from affective to cognitive empathy when working with individuals whose actions evoked personal discomfort. Instead of engaging emotionally, they adopted a more detached, intellectual understanding of the client’s perspective. This shift likely helped them manage personal discomfort while maintaining professional care. This finding echoes Bloom’s (2017) argument that empathy bias influences perceptions and treatment of others. As an alternative, compassion has been proposed as a more equitable and sustainable response to support others’ suffering. Compassion is characterized by warmth, care, and a strong motivation to help others, which increases prosocial behavior by fostering a benevolent attitude toward others (Klimecki & Singer, 2012). Supporting this view, compassion training research such as by Leiberg et al. (2011) found increased prosocial behavior and positive attitudes toward strangers while increasing positivity within oneself. Such findings suggest that cultivating compassion, rather than relying solely on empathy, may provide a more sustainable approach to therapeutic practice.

Furthermore, evidence from neuroscience suggests that adopting an other-orientated response, such as compassion, may prevent over-identification with others’ suffering while enabling the self-regulation of negative emotions triggered by empathic responses (Klimecki & Singer, 2012). For instance, studies of compassion training, particularly practices like loving-kindness meditation, have demonstrated positive effects on immune function, reduced stress, decreased negative affect, and increased positive affect (Goetz & Simon-Thomas, 2017; Klimecki & Singer, 2012; Watson et al., 2023). By fostering an other-oriented perspective, compassion allows professionals to maintain care and motivation to help without over-identifying with clients’ suffering (Bloom, 2017). Consequently, the risk of empathic distress and its associated negative outcomes may be significantly reduced.

## Implications

Overall, this study emphasizes the importance of providing adequate support and resources to those in the helping professions. Fortunately, there is hope in cultivating health and wellness to help professionals through compassion-based training practices. Integrating compassion-focused strategies into professional training and practice may mitigate the risks associated with empathic engagement (Klimecki et al., 2013). Furthermore, the study’s findings underscore the importance of organizational support in mitigating the challenges associated with empathic practice. Work health and safety regulations, such as those outlined by Safe Work Australia (2022), emphasize addressing psychosocial hazards to prevent harm. While stress is not an injury, it can cause physiological and psychological harm over time (Schauer & Elbert, 2010). Organizations can play a pivotal role by educating employees on the benefits of compassion, providing access to training programs, and fostering environments that prioritize mental health and well-being. Additionally, regular supervision, peer support systems, and workload management can help reduce the risk of burnout and empathic distress fatigue. Promoting self-care and resilience-building practices at an organizational level further reinforces individuals’ efforts to sustain emotional well-being in demanding roles.

## Limitations

IPA prioritizes detailed insights and a contextual understanding of experiences over generating broadly generalizable findings. This study restricted the context to professionals working in private practice and providing counselling under the Australian Better Access Initiative. Consequently, the findings reflect the perspective of a relatively homogenous sample, which may not fully capture the diversity of experiences present in other professional contexts.

## Conclusion

This study highlighted the dual-edged nature of empathy in therapeutic contexts, where risks to practitioner well-being counterbalance its benefits for client engagement. By emphasizing emotional regulation, integrating compassion-focused strategies, and fostering supportive organizational practices, professionals can navigate the challenges of empathic engagement more effectively. Future research should continue exploring the mechanisms underlying these phenomena, focusing on longitudinal studies and interventions to safeguard the health and effectiveness of those in the helping professions.

## Data Availability

The authors confirm that the data supporting the findings of this study are available within the manuscript.*
